# Evolution of a Cohort of COVID-19 Infection Suspects Followed-Up from Primary Health Care

**DOI:** 10.3390/jpm11060459

**Published:** 2021-05-24

**Authors:** Valle Coronado-Vázquez, Maria del Valle Ramírez-Durán, Juan Gómez-Salgado, María Silvia Dorado-Rabaneda, Elena Benito-Alonso, Marina Holgado-Juan, Cristina Bronchalo-González

**Affiliations:** 1 Group B21-20R, Health Research Institute of Aragon (IIS), 50009 Zaragoza, Spain; mvcoronado@msn.com; 2 Centro de Salud de Illescas, Servicio de Salud de Castilla-La Mancha, 45200 Toledo, Spain; mholgado@sescam.jccm.es; 3 Department of Health Sciences, Universidad Católica Santa Teresa de Jesús de Ávila, 05005 Ávila, Spain; 4 Department of Nursing, Universidad de Extremadura, 10600 Plasencia, Spain; valleramirez@unex.es; 5 Department of Sociology, Social Work and Public Health, Faculty of Labour Sciences, University of Huelva, 21071 Huelva, Spain; 6 Safety and Health Postgraduate Programme, Universidad Espíritu Santo, 092301 Guayaquil, Ecuador; 7 Consultorio Yuncos, ZBS Illescas, Servicio de Salud de Castilla-La Mancha, 45210 Toledo, Spain; silvia_dorado_r@hotmail.com (M.S.D.-R.); bronchalog@gmail.com (C.B.-G.); 8 Consultorio El Viso de San Juan, Servicio de Salud de Castilla-La Mancha, 45215 Toledo, Spain; ebenitoa@sescam.jccm.es

**Keywords:** COVID-19, epidemiology, telemedicine, teleconsultation, primary health care, management per site/severity

## Abstract

Diagnosis and home follow-up of patients affected by COVID-19 is being approached by primary health care professionals through telephone consultations. This modality of teleconsultation allows one to follow the evolution of patients and attend early to possible complications of the disease. The purpose of the study was to analyze the evolution of a cohort of patients with suspected SARS-CoV-2 disease followed by primary care professionals and to determine the factors that are associated with hospital admission. A prospective cohort study was carried out on 166 patients selected by consecutive sampling that showed symptoms compatible with COVID-19. The follow-up was approached via telephone for 14 days analyzing hospitalization and comorbidities of the patients. There were 75% of the hospitalized patients that were male (*p* = 0.002), and 70.8% presented comorbidities (*p* < 0.001). In patients with diabetes, the risk of hospitalization was 4.6-times larger, in hypertension patients it was 3.3-times, those suffering from renal insufficiency 3.8-times, and immunosuppressed patients 4.8-times (IC 95%: 1.9–11.7). In 86.7% of the cases, clinical deterioration was diagnosed in the first seven days of the infection, and 72% of healing was reached from day seven to fourteen. Monitoring from primary care of patients with COVID-19 allows early diagnosis of clinical deterioration and detection of comorbidities associated with the risk of poor evolution and hospital admission.

## 1. Introduction

The SARS-CoV-2 virus appeared in December 2019 in Wuhan, China, and spread from there globally, and was declared a global pandemic on 11 March 2020 [1].

Human coronavirus such as SARS-CoV, MERS-CoV, and SARS-CoV-2 are responsible for severe lower respiratory tract infections with extra-pulmonary manifestations secondary to a cascade of inflammation [2,3].

In the SARS-CoV-2 structure, there are glycoprotein S spikes projecting to the exterior that are the facilitators to unite to the cells and infect them [4]. Protein S uses the angiotensin-converting enzyme II (ACE II) and converts angiotensin I in angiotensin II increasing its vasoconstrictor action. This enzyme locates in mucosa cells, alveolar cells (type I and II), arteries, intestine, etc. [4]. The coronavirus has an incubation period of 5.2 days and a reproductive number between 2 and 2.5, responsible for the extreme pandemic expansion [5].

The scientific community reported that the infection’s most characteristic symptoms are fever, cough, myalgia, and fatigue. Other less common but frequently present are expectoration, headache, hemoptysis, and diarrhea. Anosmia and ageusia are also frequent and helpful in the young and women, but not determinant to diagnose COVID-19 infection [6]. Other neurological symptoms are consciousness alterations, encephalitis, and stroke [7]. SARS-CoV-2 infection can progress to an acute respiratory distress syndrome needing ICU admission and associated with a high mortality rate [8,9,10].

Most of the recent studies are based on data from hospitalized patients. However, an Israeli study from primary healthcare reported the same most characteristic symptoms: cough, fever, myalgia, and fatigue. As additional symptoms, they reported abdominal pain, thoracic pain, loss of appetite, bitter taste, and chills [11,12].

WHO defined a suspected case of COVID-19 infection as “a patient with acute respiratory illness, fever, and at least one sign/symptom of respiratory disease (e.g., cough, shortness of breath), and with no other etiology that fully explains the clinical presentation and a history of travel to or residence in a country/area or territory reporting local transmission (...) of COVID-19 disease during the 14 days prior to symptom onset. Or a patient with any acute respiratory illness and having been in contact with a confirmed or probable COVID-19 case (…) in the last 14 days prior to onset of symptoms; or a patient with severe acute respiratory infection (fever and at least one sign/symptom of respiratory disease (e.g., cough, shortness breath) and requiring hospitalization and with no other etiology that fully explains the clinical presentation” [13].

The Center for Disease Control and Prevention in China published the largest case-series report of SARS-CoV-2 infection (72,314 cases). Most of them (81%) were classified as mild (no pneumonia or mild pneumonia), 14% were severe, and 5% critical (respiratory insufficiency, septic shock, and or multiorgan failure), 87% of the cases were aged between 30 and 79 years old [14].

There has been reported in hospitalized patients some risk factors to develop a severe course of the SARS-CoV-2 infection such as being older than 65 years, living in a nursing home, having chronic pulmonary, renal, hepatic, or cardiovascular diseases, being in treatment with chemotherapy and immunosuppressors, smoking, obesity, and diabetes [9,15].

The infection’s rapid progression required multiple adaptations and organizational changes in all health systems. Among these changes, telemedicine contributes to continue the monitoring of patients from home during COVID-19 isolation or general lockdown. This monitoring is beneficial in both COVID-19 infection and chronic disease cases, respecting the social distancing measures [16,17,18,19]. The first months of the pandemic were chaotic due to a lack of diagnosis testing material. Nonetheless, primary healthcare workers were essential to detect suspected cases, isolate, and control them until they overcome the infection or showed complications [20].

This study aimed to determine the evolution of a cohort of suspected cases of COVID-19 monitored by phone in primary healthcare and to analyze associated factors to hospitalization admission based on the hypothesis that telephone monitoring from primary healthcare allowed to an early detection of complications and need for hospital admission.

## 2. Materials and Methods

### 2.1. Study Design and Participants

A longitudinal descriptive study was used to answer the purpose from March to May 2020 using a consecutive sampling to select the participants.

The inclusion criteria were: adult patients (≥18 years) consulting with their physician in Illescas’ healthcare area (Toledo) from 23 March 2020 to 20 May 2020, including those who presented symptoms of COVID-19 infection (fever, cough, dyspnea, asthenia, headache, odynophagia, arthromyalgia, tastelessness, anosmia, diarrhea, and anorexia) and/or those who reported a close contact with patients diagnosed with SARS-COV-2 infection.

Exclusion criterion: Patients from other healthcare areas with a different reference hospital.

Due to the lack of a diagnostic test, these patients were home-isolated for 14 days as a suspected COVID-19 infected. The sample size for an expected healing rate of 75%, a relative risk of 0.78, an alpha risk of 5%, and a power of 90%, adding 20% for losses, was 160 patients.

### 2.2. Data Collecting

Participants were included in the study after contacting their physician due to possible COVID-19 symptoms. The telephone follow-up was carried out after 48 h, 4, 7, 10, and 14 days and terminated whether the patient was recovered or hospitalized. In the follow-up, the recollected data was about their present clinical situation and new symptoms, performing a face-to-face consult when a poor evolution of the disease occurred (see Table S1). Sociodemographic data was also collected along with possible contact with infected patients, sort of symptoms, and comorbidities. All of the data was collected by family physicians during the patient interview and using their clinical history in five consultation rooms of the Illescas Primary Care Centre (Toledo, Spain) (see Figure S1).

### 2.3. Data Analysis

Data was analyzed using SPSS software version 24 (IBM, Armonk, NY, USA). Quantitative variables were expressed in mean, standard deviation, and percentages for the qualitative variables. To compare the categorical variables, the Chi Square test and Student’s t test were used for the quantitative ones. The relative risk of hospital admission was calculated according to the comorbidity present in the patients. A multivariate analysis was done using the Cox proportional hazards model. In all cases, the 95% confidence interval was determined.

### 2.4. Ethical Considerations

This study respected and followed the Helsinki Declaration in its recommendations for research with humans. All participants read and signed an informed consent form given to them before the study started. Researchers are engaged in maintaining the patients’ data confidentiality. This research obtained a positive evaluation from the Ethical Committee of Clinical Research of Toledo Hospital Complex.

## 3. Results

The selected cohort consisted of 166 patients with one or more COVID-19 symptoms. The mean age was 49.5 (SD = 16.9), 80.2% of the sample was younger than 64 years old. 53.9% were women, and the most frequent comorbidities were hypertension (25.5%) and COPD (7.2%). Table 1 shows all comorbidities and their frequency. Among symptoms, 50% of patients showed cough, 44.5% fever, and 26.5% asthenia. There was 48.2% of patients referred for close contact with COVID-19 diagnosed patients.

At the end of the follow-up, 85.3% of the patient had healed or improved. Hospital admission occurred in 14.7% of the cases. All patients admitted to the hospital were located on the internal medicine unit after a positive PCR detection. Nobody needed ICU admission, and nobody died during the 14 days of monitoring.

Patients showed a clinical decline in the first seven days in 86.7% of the cases, and healing occurred between the 7th and 14th day in 72% of the cases. Table 2 shows these results. A total of 36 patients needed thorax an X-ray showing viral pneumonia—52.7% of the patients. From all hospitalized patients 75% were men (*p* = 0.002) and 70.8% presented some comorbidity (*p* < 0.001).

Compared with those who were healed or had a clinical improvement, there were more hospital admissions at an older age (student t = 5.58; *p* < 0.001), among men (X2 = 9.33; *p* = 0.002), those with hypertension arterial (X2 = 11.7; *p* = 0.001), diabetes (X2 = 14.8; *p* < 0.001), chronic renal failure (X2 = 8.33; *p* = 0.004), and in those who presented immunosuppression (X2 = 6.56; *p* = 0.01). There was no significant association with the type of symptoms. The risk of hospitalization admission was higher (4.6-times) in diabetic patients, 3.3-times in hypertensive patients, 3.8-times in those with kidney failure, and 4.8-times (95% CI: 1.9–11, 7) in the immunosuppressed (Table 3). In patients over 50 years of age compared to those who are younger, having immunosuppression was a risk factor for hospital admission (hazard ratio = 6.04; 95% CI: 1.16–31.37).

## 4. Discussion

The findings confirmed that telephone follow-up by primary health workers was relevant in patients who were possibly COVID-19 infected during the first pandemic months in Spain, enabling prior detection, treatment, and being able to foresee possible complications.

Comorbidities such as diabetes, arterial hypertension, or chronic renal insufficiency in the presence of an infection by the SARS-CoV-2 virus put patients at greater risk of worse clinical evolution with consequent hospitalization admission. These findings agree with other prior studies [21,22,23]. In Chung et al. [24], hospitalized COVID-19 infected patients with diabetes mellitus developed high levels of inflammation biomarkers and a higher risk of suffering complications, including mortality in the 28 days into infection evolution. Zhou et al. [25] reported that even though diabetes did not elevate the mortality risk, there were many diabetic patients in the ‘non-surviving’ group. Accordingly, in the study of Wu et al. [14] neither was there any relation between diabetes and mortality. However, there was a greater possibility of developing acute respiratory distress syndrome after 40 days of hospitalization follow-up. In Cummings et al. [26], monitoring and analysis of critical hospitalized patients showed that 63% of them had hypertension as comorbidity. The above findings set us towards more focused attention on patients with such comorbidities due to the reportedly high risk of developing complications. This attention and monitoring should preferable developed in primary healthcare where they are still at home and healthier.

Patients from our studied cohort who manifested clinical worsening showed it in the first seven days of the infection diagnosis (86.7%). Agreeing with these results, the studied cohort from Zhang et al. [27] also reported the same seven days from symptoms appearance until hospitalization admission, not finding statistical difference among age groups. Therefore, it is the first seven days after the onset of the infection when patient monitoring is of great interest.

In this study, 70% of patients presented infection overcome from the seventh day forward until the fourteenth. Consequently, domiciliary telephone monitoring from primary healthcare represents a valuable, safe, and resources friendly option to evaluate patients’ evolution. Furthermore, it avoids unnecessary visits to healthcare centers enabling the required isolation restrictions [28,29,30,31].

Favorable clinical evolution resulted in the overcoming of the infection or clinical amelioration which was shown in almost the entire cohort (85.3%), agreeing with the findings from Pallarés et al. [32]. This may be explained regarding the cohort’s mean age of 49.5 years, in whose COVID-19 symptoms are usually mildly presented. The results from Zhang et al. [19] showed mild and moderate COVID-19 symptoms in 50% of the patients from their cohort of 185 patients under 45 years old. Although the mean age might have influenced the rate of mild COVID-19 presentations and the overcome of the infection, the factors with higher association to a poor evolution were those related to chronic diseases.

Hospital admission was more frequent in men (75% of the admitted cases) which was also reported in Cummings et al. [18], Wiersinga et al. [33], Jin et al. [34], and Siso Almirall et al. [35]. COVID-19 clinical presentations in this cohort were similar to those reported in other studies, showing a stabilization of the clinical manifestations of the infection [33,34,35].

Patients telephone monitoring has been proven to be a beneficial tool in COVID-19 response similarly for other diseases, and it has been implemented in many countries [28,30,31,36]. Specially in Italy, one of the European countries where Covid-19 struck first and hardly, the role of family medicine and telemedicine was found to be essential in the prevention of patients’ referral to the emergency departments, thus avoiding the collapse of hospitals and primary centers and facilitating the first triage and hospital admission of those patients that were likely to have a critical deterioration due to comorbidities and a strong illness evolution [36]. Actually, those patients suspected or at high risk for COVID-19 infection, unless with moderate disease, were screened and treated home initially [36]. Bokolo [28] explains in his review some factors that impact the adoption of telemedicine such as inadequate training or lack of regulation and advocacy. However, his findings agree with the consideration of telemedicine as a helpful tool to control the spread of COVID-19. Imlach et al. [30] obtained positive results from the use of telemedicine in primary healthcare during the lockdown period, reaching high levels of patient’s satisfaction in areas such as personal security, attention quality, and accessibility. However, limitations remained when a physical exploration was needed. Crane et al. [31] implemented telephone and videoconference monitoring in patients waiting for the PCR result and after obtaining it. The videoconference was the most used option. Patient monitoring from primary healthcare creates data that allows the analysis of the SARS-CoV-2 infection evolution and its long-term consequences [29].

This study showed some limitations such as the selected period of monitoring. Some research reported symptoms ahead of the fourteenth day. In Carvalho-Schneider et al. [37] two-thirds of the analyzed patients with mild COVID-19 symptoms manifestations remained well after two months of the infection setting. The symptoms were anosmia, ageusia, dyspnea, or asthenia. This information suggests the necessity of an increased monitoring period to study and analyze the persistent manifestations of the infection.

This study was developed in primary healthcare where patients with immunosuppression are less prevalent, and it explains the fewer patients with this pathology. These findings suggest the need for more studies with a larger sample, including immunosuppression and COVID-19.

Another limitation of the study is the lack of diagnosis material and the overflow of patients that clinicians in primary healthcare suffered in the early period of the pandemic setting. This fact explains that our cohort was created of suspected cases of COVID-19 instead of confirmed cases. Recently, the Spanish healthcare system can affront the COVID-19 more accurately thanks to diagnosis tests and protective equipment availability, vaccination strategies, and the precise monitoring of patients with comorbidities. In fact, as same as happened in near countries [36], new tendencies that arrived with the COVID-19 pandemic have been implemented in our daily practice, i.e., the use of telemedicine in Primary Care and Hospital consultations, the reorganization of hospital spaces, or the adoption of more flexible policies for the recruitment of healthcare staff and the increase of the professional-patient ratio. However, the primary healthcare approach of the COVID-19 has not been widely reported, making these findings a contribution to the first steps of developing clinical knowledge.

## 5. Conclusions

In conclusion, it has been stated that the risk of hospital admission in patients with suspected COVID-19 infection is higher in those with comorbidities such as diabetes, hypertension, renal insufficiency, and or immunosuppression. Telephone monitoring from primary healthcare allows the evaluation of the patient’s evolution and enables the early detection of complications, knowledge, and treatment of those comorbidities that increase the risk of hospitalization.

## Figures and Tables

**Table 1:** Comorbidities proportion presented in patients.

Comorbidities	*n* (%)
Hypertension	42 (25.5)
COPD	12 (7.2)
Diabetes	11 (6.6)
Dyslipidemia	10 (6)
Cerebrovascular disease	8 (4.8)
Cardiovascular disease	6 (3.6)
Cancer	3 (1.8)
Chronic renal disease	8 (4.8)
Immunosuppression	3 (1.8)

**Table 2:** Patients’ clinical evolution during the 14 days phone monitoring.

	Clinical Stability	Clinical Deterioration	Hospital Admission	Healing
48 h *n* (%) *n* = 165	139 (84.24)	16 (9.6)	2 (1.2)	8 (4.8)
4 days *n* (%) *n* = 152	110 (72.3)	16 (10.5)	3 (1.9)	23 (15.1)
7 days *n* (%) *n* = 123	76 (61.7)	14 (11.3)	4 (3.2)	29 (23.5)
10 days *n* (%) *n* = 85	46 (54.1)	5 (5.8)	3 (3.5)	31 (36.4)
14 days *n* (%) *n* = 49	26 (53.1)	2 (4.1)	1 (2)	20 (40.8)

**Table 3:** Associated comorbidities to hospital admission.

	Relative Risk (95% CI)	*p*
Immunosuppression	4.8 (1.9–11.7)	0.01
Diabetes	4.6 (2.3–9.2)	0.0001
Chronic renal insufficiency	3.8 (1.7–8.6)	0.003
Arterial hypertension	3.3 (1.6–6.9)	0.001

## Data Availability

All data is available within this article and its artwork.

## Supplementary Materials

The following are available online at https://www.mdpi.com/article/10.3390/jpm11060459/s1, Table S1: Annex 1. Data Collection Sheet; Figure S1: Participants’ flowchart.
