# Supplementary material for: Evolution of a Cohort of COVID-19 Infection Suspects Followed-Up from Primary Health Care
Source: J Pers Med. 2021 May 24;11(6):459. doi: 10.3390/jpm11060459 (PMC8224796; doi:10.3390/jpm11060459)
Supplement: Supplementary file 1 [file jpm-11-00459-s001.zip › Table S1. Data collection sheet.pdf]

Table S1. Annex 1: Data Collection Sheet

**Patient identification**	Initials									
**Data of enrolment in the study**	dd-mm-yy									
**Age**	Years of age									
**Sex**	Male	Female								
**Contact with any COVID-19 patient**	No	Yes								
**Chronic diseases**										
**COVID-19 related symptoms**	No	Yes								
**Data onset symptoms**	dd-mm									
**Sort of symptoms**	Fever	Cough	Fatigue	Dyspnoea	Odynophagia	Headache	Arthromyalgia	Anosmia-Ageusia	Diarrhoea	Other
**48 hours evolution**	Healed	Clinical deterioration	Hospital admission	Deceased						
**48 hours symptoms**	Fever	Cough	Fatigue	Dyspnoea	Odynophagia	Headache	Arthromyalgia	Anosmia-Ageusia	Diarrhoea	Other
**4 days evolution**	Healed	Clinical deterioration	Hospital admission	Deceased						
**4 days symptoms**	Fever	Cough	Fatigue	Dyspnoea	Odynophagia	Headache	Arthromyalgia	Anosmia-Ageusia	Diarrhoea	Other

**7 days evolution**	Healed	Clinical deterioration	Hospital admission	Deceased						
**7 days symptoms**	Fever	Cough	Fatigue	Dyspnoea	Odynophagia	Headache	Arthromyalgia	Anosmia-Ageusia	Diarrhoea	Other
**10 days evolution**	Healed	Clinical deterioration	Hospital admission	Deceased						
**10 days symptoms**	Fever	Cough	Fatigue	Dyspnoea	Odynophagia	Headache	Arthromyalgia	Anosmia-Ageusia	Diarrhoea	Other
**14 days evolution**	Healed	Clinical deterioration	Hospital admission	Deceased						
**14 days symptoms**	Fever	Cough	Fatigue	Dyspnoea	Odynophagia	Headache	Arthromyalgia	Anosmia-Ageusia	Diarrhoea	Other
**Date of Hospital admission**	dd-mm									
**Type of admission**	Hospital unit	ICU								
**Loss during monitoring. Causes.**										
