# Supplementary figures and images for: Evolution of a Cohort of COVID-19 Infection Suspects Followed-Up from Primary Health Care
Source: J Pers Med. 2021 May 24;11(6):459. doi: 10.3390/jpm11060459 (PMC8224796; doi:10.3390/jpm11060459)

## Participants' flowchart

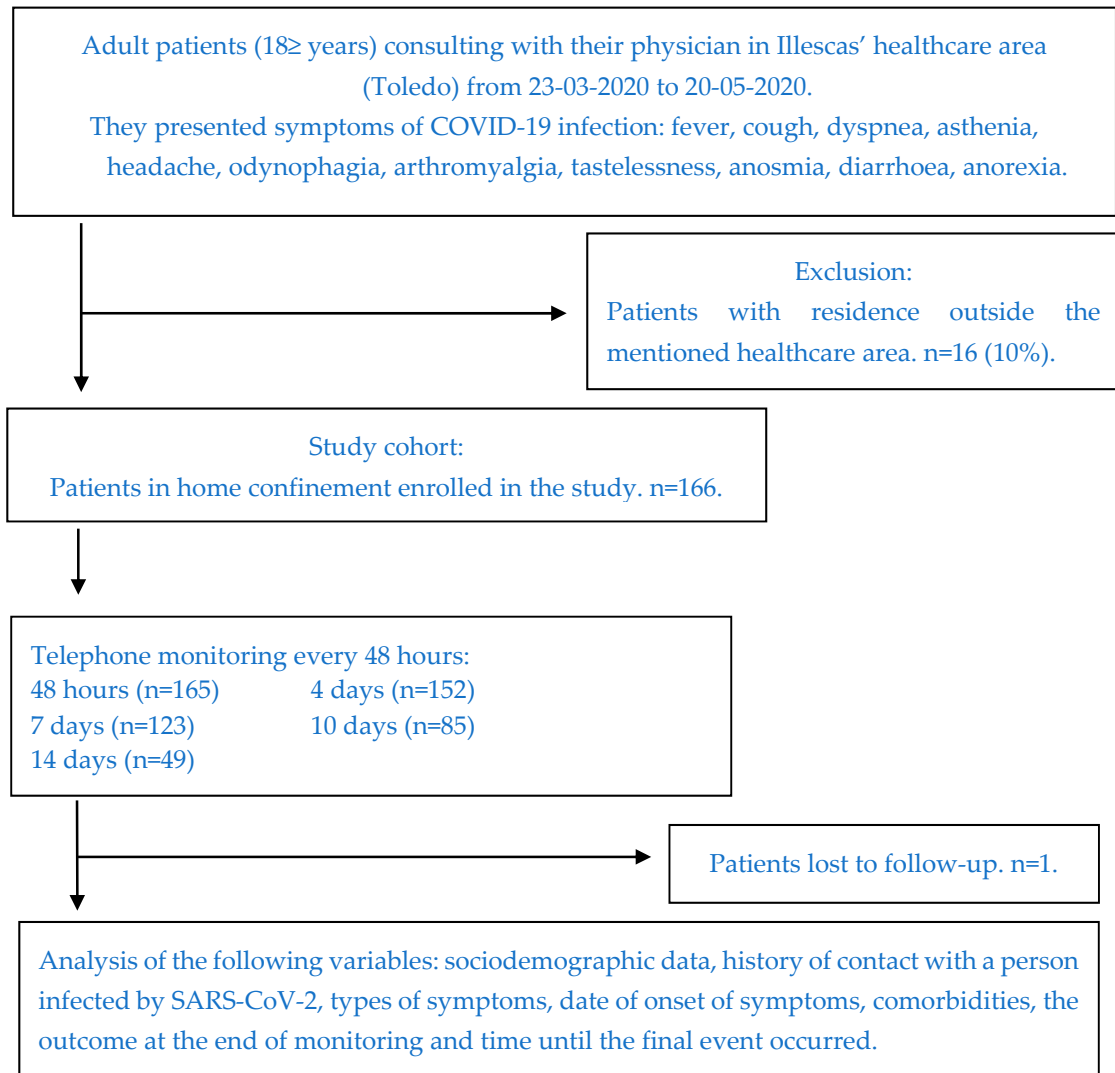

Supplement: Supplementary file 1 [file jpm-11-00459-s001.zip › Figure S1. Participants flowchart.pdf]
